# Happier during lockdown: a descriptive analysis of self-reported wellbeing in 17,000 UK school students during Covid-19 lockdown

**DOI:** 10.1007/s00787-021-01934-z

**Published:** 2022-02-17

**Authors:** Emma Soneson, Stephen Puntis, Nikki Chapman, Karen L. Mansfield, Peter B. Jones, Mina Fazel

**Affiliations:** 1 Department of Psychiatry, University of Cambridge, Douglas House, 18b Trumpington Road, Cambridge, CB2 8AH UK; 2 Department of Psychiatry, Warneford Hospital, University of Oxford, Oxford, OX3 7JX UK; 3 Fresh+ Group, Fresh CAMHS Liverpool, Catkin Building, Alder Hey Children’s NHS Foundation Trust, Eaton Road, Liverpool, L12 2AP UK

**Keywords:** Mental health, Wellbeing, Young people, School, Covid-19, Lockdown

## Abstract

Relatively little research has focused on children and young people (CYP) whose mental health and wellbeing improved during Covid-19 lockdown measures. We aimed to (1) determine the proportion of CYP who self-reported improvement in their mental wellbeing during the first Covid-19 lockdown and (2) describe the characteristics of this group in relation to their peers. We conducted a descriptive analysis of data from the 2020 OxWell Student Survey, a self-report, cross-sectional survey of English CYP. A total of 16,940 CYP primarily aged 8–18 years reported on change in mental wellbeing during lockdown. We characterised these CYP in terms of school, home, relational, and lifestyle factors as well as feelings about returning to school. One-third (33%) of CYP reported improved mental wellbeing during the first UK national lockdown. Compared with peers who reported no change or deterioration, a higher proportion of CYP with improved mental wellbeing reported improved relationships with friends and family, less loneliness and exclusion, reduced bullying, better management of school tasks, and more sleep and exercise during lockdown. In conclusion, a sizeable minority of CYP reported improved mental wellbeing during lockdown. Determining the reasons *why* these CYP felt they fared better during lockdown and considering *how* these beneficial experiences can be maintained beyond the pandemic might provide insights into how to promote the future mental health and wellbeing of school-aged CYP. All those working with CYP now have an opportunity to consider whether a systemic shift is needed in order to understand and realise any learnings from experiences during the pandemic.

**Supplementary Information:**

The online version contains supplementary material available at 10.1007/s00787-021-01934-z.

## Introduction

The extensive and indiscriminate societal changes during the Covid-19 pandemic have significantly impacted the lives of children and young people (CYP). Whilst the rates of infection and public health responses to the pandemic have differed across time and place, widespread school closures and social distancing measures have interrupted CYP’s education and disturbed their social milieu across the globe, altering the way they interact with those around them. Several studies have offered insight into the impact the pandemic has had on CYP’s mental health and wellbeing, mostly reporting on its negative mental health consequences. For example, in July 2020, as public health measures from the first United Kingdom (UK) lockdown were starting to ease and the school year was ending, the English national survey of approximately 3500 CYP showed that one in six CYP aged 5–16 years had a clinically diagnosable mental health disorder, representing an increase from one in nine in 2017 [1]. Evidence from a longitudinal study of approximately 60,000 young people aged 13–18 years in Iceland also demonstrated increases in average levels of depressive symptoms and decreases in mental wellbeing during September–November 2020, when the country had strict physical distancing measures and most schools were limited to online provision, as compared with 2016 and 2018 [2]. Smaller studies from England, the Netherlands, the United States, and Australia that have compared pre-pandemic data with data collected in March–May 2020, when each country was experiencing lockdown measures and school closures, also provide evidence for deterioration in various domains of CYP’s mental health [3–6].

This picture of deterioration is, however, far from uniform. Some studies have found little to no change in average levels of CYP’s mental health or report deterioration only in select domains [3, 7]. Of note, two studies have reported improvement in the average levels of CYP’s mental health and wellbeing during the pandemic. First, Widnall and colleagues [8] followed a group of approximately 1000 English 13- to 14-year-olds from October 2019 to April/May 2020, when the UK had strict lockdown measures and school closures. The authors found an increase in wellbeing and decrease in risk of anxiety, with the greatest improvements for those who had poor pre-pandemic mental health and wellbeing and low connectedness with school, peers, and family. Second, Penner and colleagues [9] reported improved mental health in a sample of over 300 American 10–14-year-olds followed from January 2020 to April/May 2020, when the country had stay-at-home measures and schools were limited to online provision. The 20% of their sample who had poor mental health pre-pandemic experienced clinically significant improvements in internalising, externalising, and total problems scores. Among the rest of the sample, there were statistically (though not clinically) significant improvements in internalising and total problems.

Furthermore, within several of the studies that showed an *average* deterioration in levels of mental health and wellbeing, there have been subgroups of CYP who either improved or did consistently well during the pandemic. In the English national survey (described above), just over one-quarter of CYP aged 11–16 years reported that lockdown had made their life better [1]*.* Findings from the UK Co-SPACE study, which includes repeated data from approximately 3000 parents and CYP (with no pre-pandemic data), also highlighted subgroups of CYP who had consistently lower-than-average symptoms of conduct, hyperactivity, and emotional problems in the first months of the pandemic (March–July 2020), and, in the case of emotional problems, a sub-group that improved over time [10]. Another cross-sectional study of approximately 1000 Canadian CYP aged 6–18 years in April–June 2020, during Canada’s emergency public health measures including school closures, found that 20% had experienced a parent-reported improvement in at least one of six domains of mental health: depression, anxiety, irritability, attention, hyperactivity, and obsessions/compulsions [11]. Among participants aged 10–12 years, 9–13% self-reported improvements across five domains of mental health, as did 8–20% of those aged 13–18 years. For both age groups, the greatest improvements were in the depression and anxiety domains. Improvements in these domains were associated with having a pre-pandemic psychiatric diagnosis and less stress from social isolation.

In addition to quantifying improvements in mental health and wellbeing, it is important to better understand some of the reasons behind these improvements. A number of qualitative studies conducted in the UK, United States, Ireland, and Portugal have explored CYP’s experiences of the pandemic between May and October 2020, during which time there was significant variation in Covid-related public health measures and restrictions [12–15]. Whilst most of the narratives in these studies were primarily centred around negative impacts (e.g. disruptions to schooling or social networks, deterioration in mental health), some CYP cited positive changes. These changes were related to school (e.g. enjoyment of self-directed learning [12], less schoolwork, less stress and pressure from school and activities [15]), relationships (e.g. strengthened family bonds [15, 16], avoiding unwanted interactions at school [15]), and having more autonomy over their schedule and activities (e.g. more time to pursue hobbies [12, 14, 15]). Stallard and colleagues [17] specifically aimed to explore positive changes related to the pandemic. In their survey of 385 parents and carers with children aged 6–16 years in Portugal and the UK, conducted during each country’s first lockdown in May–June 2020, the authors asked participants to list ‘any positives’ to come out of the pandemic and social distancing measures. Nearly nine in ten provided a response, which fell under categories related to interpersonal relationships (e.g. improved relationships with family), appreciation of life (e.g. opportunity to assess personal values, ability to live a healthier life), new possibilities (e.g. learning new skills, better work-family balance, changes to their children’s education), and spiritual growth (e.g. positive social and environmental changes). Whilst the answers were from a parent/carer perspective, many responses related to the family or home environment, and, therefore, of likely relevance to CYP.

Overall, there has been insufficient focus on CYP who have experienced improved mental health and wellbeing during the pandemic, especially in terms of studies that have analysed data gathered directly from CYP. Identifying the specific factors that may have contributed to improved mental health and wellbeing provides the opportunity to understand how we can create lasting positive change to promote better mental health and wellbeing post-pandemic [18], which might have impact beyond just those CYP who have reported improvements in lockdown. In this study, we aimed to (1) determine the proportion of CYP who self-reported improvement in their mental wellbeing during the first Covid-19 lockdown and (2) describe the characteristics of this group in relation to their peers.

## Methods

### Data source: OxWell Student Survey

This study used data from the OxWell Student Survey, a recurring, cross-sectional, self-report survey that includes variables relating to CYP’s mental health and wellbeing [19–21]. The survey contains a set of ‘core’ questions repeated in each survey iteration as well as new questions added in response to social and environmental events (e.g. the Covid-19 pandemic) and emerging research hypotheses. Students, parents, teachers, and health commissioners provide input on the survey to ensure its quality and relevance [21].

### Context: 2020 data collection

For this analysis, we used data collected in June–July 2020, during the first round of partial school closures due to the Covid-19 pandemic. On 26 March 2020, the first national lockdown came into effect. Lockdown measures included a ‘stay at home’ order and closure of all non-essential businesses and organisations. Measures also included school closures for all CYP except those whose parents were classified as essential (key) workers (e.g. health care workers, law enforcement, and others responsible for delivering the Covid-19 public health response) and those considered ‘vulnerable’ (e.g. CYP under the care of statutory/social services because of family difficulties, CYP with mental health concerns, and CYP in families or social situations deemed by schools to be of concern and, therefore, in need of support over the lockdown) [22]. From 1 June 2020, CYP in year groups preparing for transitions or national exams (English school years 6, 10, and 12) were also eligible to receive in-school provision [19]. Not all CYP eligible for an in-school place used the place, for a variety of reasons, for example, that they were able to be cared for at home by other family members or they did not like the school experience, which would have been very different to their usual schooling. CYP not in school received at-home education provision, which varied widely across schools and households [19]. It is estimated that around 10–15% CYP were actually attending in-school provision during the survey period [23].

### Participants

Mainstream primary and secondary schools and further education colleges (FECs) in the south of England were eligible to participate. Our analysis includes students in Years 4–13 (primarily aged 8–18 years) at state-maintained and independent primary and secondary schools and FECs located primarily in Oxfordshire, Berkshire, Buckinghamshire, and Gloucestershire.

### Recruitment

In May to July 2020, local authorities sent e-mails to schools in their catchment areas to invite them to participate in the study. The OxWell team sent study information to the schools that registered to participate, which then sent study information to parents. Schools enrolled students under the age of 16 years using a parental opt-out model, and those aged 16 years and older gave their own active, informed consent to participate. Schools provided students with secure log-in details where they could read about the survey and provide assent (all students) and full consent (students aged ≥ 16 years) before participating. This is described in greater detail in the study protocol [21].

### Survey measures

#### Measure selection

To ensure that we included the most relevant and meaningful variables available within the OxWell Survey in our analyses, we consulted with parents and CYP to understand what factors they thought might influence whether a student experienced improved mental wellbeing during lockdown. To this end, we created a questionnaire (the ‘stakeholder questionnaire’) for CYP who believed their mental health and wellbeing had improved during lockdown and parents who perceived improvement in their child. The stakeholder questionnaire asked for up to three reasons why participants thought their/their child’s mental health and wellbeing had improved. We shared the stakeholder questionnaire with twelve parent networks across the country and on social media (Twitter and Facebook) and received approximately 400 responses (around three-quarters from parents and one-quarter from CYP). Members of the study team (ES and NC) categorised all of the reasons provided into themes and discussed them with nine members of the Rethinking Education Action Group (https://rethinkingeducation.org.uk). This group was started by NC, a parent support group facilitator and parent expert by experience, and is made up of parents and practitioners working to ensure that mental health and wellbeing are an intrinsic part of education. These group members clarified and expanded upon the suggestions and themes from the stakeholder questionnaire.

We then compared the list of suggestions from the stakeholder questionnaire to the list of variables available in the OxWell Survey data and looked for ‘matches’ across the two lists (see Supplementary Table 1). For example, one common suggestion from the stakeholder questionnaire was that CYP’s mental health and wellbeing had improved during lockdown because there was less or no bullying. Variables available in the OxWell Survey that related to this suggestion included frequency of bullying in the past year, change in bullying over lockdown, safety at school, frequency of feeling left out, change in feeling left out during lockdown, and feelings about seeing classmates/peers when returning to school. We included all such ‘matches’ in our analyses, although it is important to note that not all suggestions provided had matching variables in the survey: for example, we were not able to explore a suggestion about not having to wear school uniform.

Supplementary Table 2 provides a full list of survey questions, their wordings, and response options used in this study, and full variable guides are available on the OxWell Study page on the Open Science Framework (OSF) website (https://osf.io/sekhr/).

#### Identifying students who reported better mental wellbeing during lockdown

We measured perceived change in mental wellbeing with the question ‘During lockdown, how happy have you been feeling in general (your mental well-being)?’. Students responded on a sliding scale of 0–100 with five labelled categories, ‘Much worse’, ‘Slightly worse’, ‘The same’, ‘Slightly better’, and ‘Much better’, anchored at scores 0, 25, 50, 75, and 100, respectively. We simplified the scale in this analysis by transforming it into groups of those doing ‘worse’ (scores 0–37.5 on the sliding scale), ‘the same’ (scores 37.6–62.5), or ‘better’ (scores 62.6–100) (*N.B.* categories are not evenly divided due to how the scale is presented in the survey; please see https://osf.io/fpbt3/ for an example of the survey scales).

#### Sociodemographic characteristics

We included the following sociodemographic measures: gender (measured as male or female or boy or girl, depending on age group), key stage (referring to students’ year in school), free school meal eligibility, immigration status (measured as student and both parents born in the UK; both parents born in the UK, student born elsewhere; student born in the UK, at least one parent born elsewhere; or student and at least one parent born elsewhere), and garden access (no access to a garden, having access to a garden but not using it, having access to a garden and using it sometimes, or having access to a garden and using it every day).

#### Student experiences of schooling, home life, lifestyle factors, and relationships

We also included the following measures in our analyses: school attendance during lockdown, perceptions of safety at school and at home, concern about school performance, academic support at school and at home, school task management, bullying, friend and family relationships, loneliness and exclusion, exercise, and sleep. We conceptually divided these measures into *reference* measures (those that asked students about their general experiences, e.g. ‘How well do you get along with your friends?’) and measures of *change during lockdown* (those that asked students how lockdown has changed their experiences, e.g. ‘During lockdown, have you got along less well, the same or better with your friends?’).

We further included measures of students’ feelings about eight aspects related to returning to school after lockdown: seeing friends again, seeing other classmates/peers, schoolwork, attending lessons, being away from home, sports and exercise activities, other school/outside-school clubs, and travelling to and from school.

#### Mental health & wellbeing

We measured mental wellbeing at the time of completing the survey using the Warwick-Edinburgh Mental Wellbeing Scale (WEMWBS) [24]. The scale has 14 positively-worded items with four response categories according to frequency (1—‘none of the time’, 2—‘rarely’, 3—‘some of the time’, 4—‘often’, 5—‘all of the time’) that sum to a total score of 14–70, with higher scores indicating higher wellbeing. The WEMWBS has demonstrated good internal consistency (*α* = 0.87) in young people aged 13–16 years [25]. In the OxWell Survey, the scale was slightly modified such that participants rated statements on a 0–100 sliding scale with the same labels as the original scale. For adolescents in Years 8–13, we also measured mental illness using the (unmodified) 25-item version of the Revised Child Anxiety and Depression Scale (RCADS) [26]. All statements have four response categories according to frequency (0—‘never’, 1—‘sometimes’, 2—‘often’, 3—‘always’). Responses sum to a Total Anxiety and Depression score (0–75), as well as a Total Anxiety sub-score (0–45) and a Total Depression sub-score (0–30), with higher scores indicating greater mental health difficulties. In this study, we used *t* scores for the Total Anxiety and Depression score [27]. The RCADS-25 has demonstrated acceptable to excellent internal consistency (subscale range *α* = 0.79 to 0.94) in school-based samples and adequately discriminates between young people with and without mental health diagnoses [26].

### Analysis

We described the aggregate characteristics of participating schools by means and standard deviations (SDs) for continuous variables, and absolute and relative frequencies for categorical variables. Similarly, we summarised students’ sociodemographic characteristics and their answers to the questions about school, home, relationships, and lifestyle using the appropriate absolute and relative frequencies, medians, and interquartile ranges (IQRs).

For our main analysis, we grouped students into one of our derived three-group self-reported wellbeing categories of doing ‘better’, ‘the same’, or ‘worse’ and compared these groups by medians, IQRs, and absolute and relative frequencies. For the questions measuring students’ feelings about returning to school after lockdown, we excluded students who reported physically attending school ‘most days’ or ‘every day’ during the lockdown. All analyses were conducted using R version 4.0.5 (using packages *tidyverse, patchwork, and summarytools*).

## Results

22,336 students across 144 primary schools, 84 secondary schools, and 7 FECs accessed the survey (Table 1). Whilst it is not possible to calculate response rate due to uncertainty around the total number of students invited, some students will not have accessed the survey for reasons including (1) not being contacted/invited by their school, (2) being opted-out by parents, (3) not reading the survey information, or (4) choosing not to participate (numbers per category are not available). Survey administrators further removed 4,731 responses (21%) due to spending fewer than 10 min on the survey or providing unrealistic/inconsistent responses. Of the 17,605 remaining participants, 16,940 answered the question about change in mental wellbeing and were included in our final sample.

**Table 1:** Aggregate characteristics of participating schools

Characteristics	
*School context*	
Urbanicity *N (%)*	
Rural	54 (23.7%)
Urban	174 (76.3%)
Missing	7 (2.9%)
Area-level deprivation decile (IMD) *N (%)*	
1–5 (most deprived)	68 (28.9%)
6–10 (least deprived)	160 (68.1%)
Missing	7 (2.9%)
Characteristics of school community*	
Percentage of pupils eligible for free school meals *Mean (SD)*	10.1 (7.4)
Percentage of pupils who are White British *Mean (SD)*	67.8 (25.6)
*Operational features of the school*	
Type of school *N (%)*	
State-funded primary	142 (60.4%)
State-funded secondary	79 (33.6%)
Independent school	7 (2.9%)
Further Education College	7 (2.9%)
*Mixed or single sex school N (%)*	
Mixed	213 (90.6%)
Female only	9 (3.8%)
Male only	6 (2.5%)
Missing	7 (2.9%)

*Data not available for independent/academy schools or FECs

### Proportion of CYP who self-reported improvement in their mental wellbeing during lockdown

**Fig. 1:**
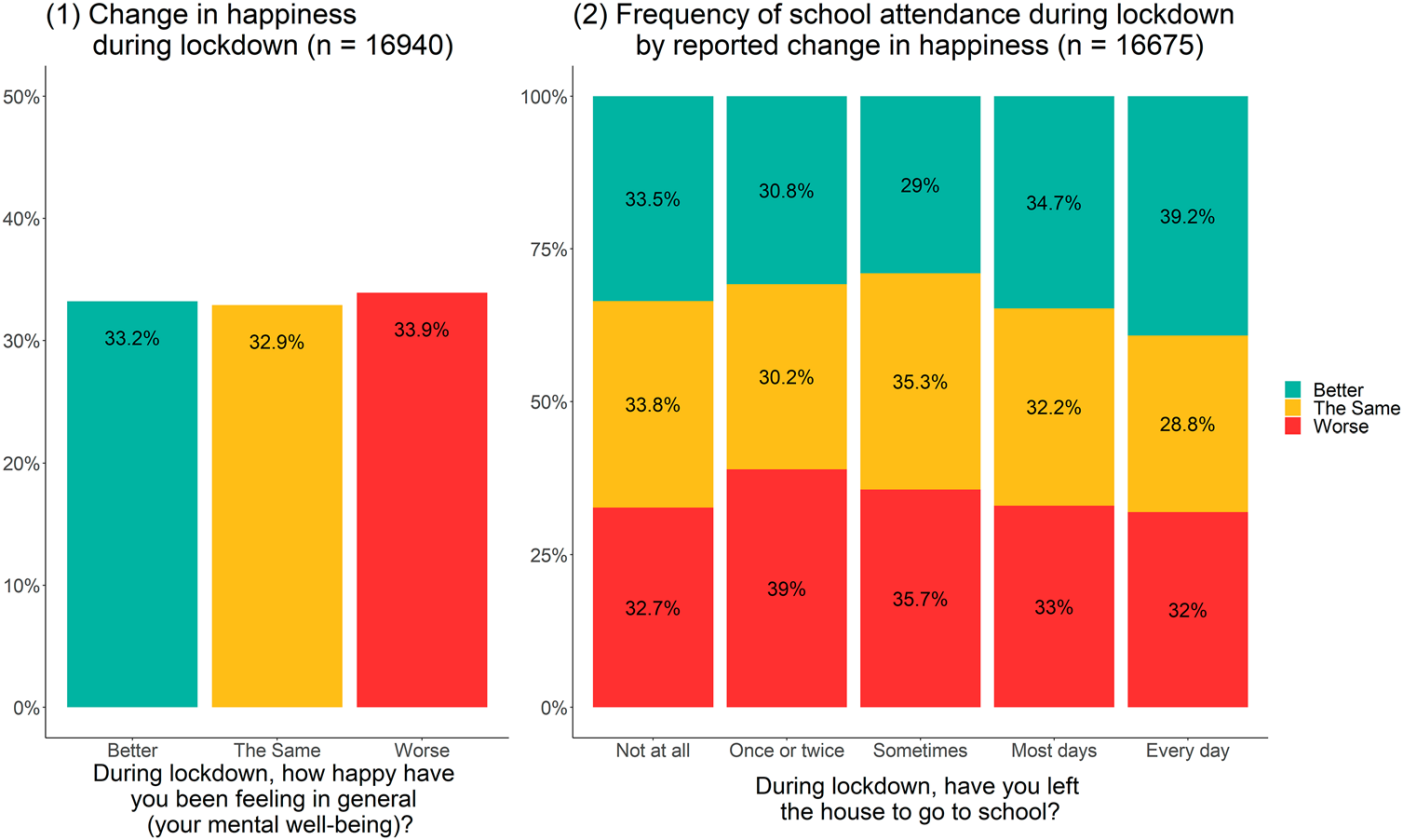
(1) Self-reported change in mental wellbeing during lockdown; (2) Frequency of school attendance by selfreported change in mental wellbeing during lockdown

One-third (*N* = 5616; 33.2%) of students reported that their mental wellbeing had improved during lockdown, compared with *N* = 5581 (32.9%) who reported that it remained the same and *N* = 5743 (33.9%) who reported that it had deteriorated (Fig. 1). Supplementary Fig. 1 shows the association between self-reported change in mental wellbeing and (1) WEMWBS and (2) RCADS scores. WEMWBS scores indicated that those who reported improved mental wellbeing during lockdown also had higher WEMWBS scores (median 53.0, IQR: 48.0; 58.0) than their peers who reported a deterioration (median 40.0, IQR: 34.0; 47.0) or no change (median 51.0, IQR: 45.0; 56.0). Those who reported improvement had lower levels of depression and anxiety (RCADS median 42.7, IQR: 36.1; 52.9) than their peers who reported deterioration (median 58.8, IQR: 48.1; 71.3) but were similar to their peers who reported no change (median 42.5, IQR: 35.6; 51.8).

### Characteristics of CYP who self-reported improvement in their mental wellbeing during lockdown

Table 2 overviews relationships between all variables of interest and self-reported change in mental wellbeing during lockdown.

**Table 2:** C﻿haracteristics by self-reported change in mental wellbeing

Variable	Missing data (%)	Worse *N (%)*	The same *N (%)*	Better *N (%)*
**Sociodemographic factors**				
*Gender*	0.6			
Male		1836 (32.2)	2361 (42.5)	2400 (43.0)
Female		3861 (67.8)	3199 (57.5)	3185 (57.0)
*Ages (key stage (KS))*	0			
Ages 8–11 (KS2)		942 (16.4)	1209 (21.7)	1276 (22.7)
Ages 12–14 (KS3)		2643 (46.0)	2983 (53.4)	2874 (51.2)
Ages 15–16 (KS4)		1293 (22.5)	919 (16.5)	1015 (18.1)
Ages 17–18 (KS5)		865 (15.1)	470 (8.4)	451 (8.0)
*Free school meal eligibility*	3.7			
No		4127 (73.9)	3972 (74.1)	3989 (73.6)
Yes		480 (8.6)	407 (7.6)	504 (9.3)
Don't know		975 (17.5)	983 (18.3)	928 (17.1)
*Immigration status*	3.2			
Student & parents all UK-born		3592 (64.3)	3343 (61.6)	3171 (58.5)
Student UK-born with at least one parent born elsewhere		1406 (25.2)	1451 (26.8)	1536 (28.3)
Both parents UK-born but not student		61 (1.1)	65 (1.2)	59 (1.1)
Student and at least one parent born elsewhere		531 (9.5)	564 (10.4)	657 (12.1)
*Garden access*	0.6			
Yes, use every day		2178 (38.1)	2612 (47.1)	2724 (48.8)
Yes, sometimes use		2905 (50.8)	2513 (45.3)	2387 (42.8)
Yes, but do not use		422 (7.4)	238 (4.3)	268 (4.8)
No		213 (3.7)	183 (3.3)	204 (3.7)
**School factors**				
*School attendance*	1.6			
Not at all		3770 (66.9)	3890 (70.5)	3865 (70.0)
Once or twice		939 (16.7)	728 (13.2)	742 (13.4)
Sometimes		417 (7.4)	413 (7.5)	339 (6.1)
Most days		328 (5.8)	320 (5.8)	345 (6.3)
Every day		185 (3.3)	167 (3.0)	227 (4.1)
*Safety at school*	2.1			
Very safe		1530 (27.2)	2067 (37.5)	2147 (38.9)
Safe		2568 (45.7)	2513 (45.5)	2390 (43.3)
Neither safe nor unsafe		1115 (19.8)	804 (14.6)	785 (14.2)
Unsafe		332 (5.9)	99 (1.8)	155 (2.8)
Very unsafe		79 (1.4)	35 (0.6)	40 (0.7)
Concern about school performance*	2.9			
Not at all worried		122 (3.2)	294 (9.5)	303 (9.7)
Not very worried		408 (10.8)	808 (26.2)	764 (24.4)
Quite worried		601 (15.9)	792 (25.6)	729 (23.3)
Worried		1457 (38.5)	873 (28.3)	901 (28.8)
Extremely worried		1198 (31.6)	322 (10.4)	436 (13.9)
*Academic support at home*	3.8			
All of the help I need		950 (17.1)	1682 (30.9)	1829 (33.7)
Most of the help I need		1414 (25.5)	1665 (30.6)	1811 (33.3)
Just about enough help		1559 (28.1)	1480 (27.2)	1186 (21.8)
Not enough help		806 (14.5)	337 (6.2)	315 (5.8)
No help at all		811 (14.6)	277 (5.1)	290 (5.3)
*Academic support at school*	3.9			
All of the help I need		739 (13.4)	1359 (24.9)	1398 (25.8)
Most of the help I need		1875 (33.9)	2042 (37.5)	2138 (39.4)
Just about enough help		1644 (29.7)	1601 (29.4)	1309 (24.1)
Not enough help		1076 (19.5)	386 (7.1)	481 (8.9)
No help at all		196 (3.5)	62 (1.1)	101 (1.9)
*School task management (change during lockdown)*	4.2			
Much better		281 (5.0)	373 (6.9)	930 (17.3)
Slightly better		688 (12.3)	875 (16.2)	1359 (25.3)
The same		1205 (21.6)	2375 (43.9)	1601 (29.8)
Slightly worse		2173 (38.9)	1500 (27.7)	1196 (22.2)
Much worse		1243 (22.2)	286 (5.3)	292 (5.4)
**Home factors**				
*Safety at home*	1.1			
Very safe		2914 (51.4)	3777 (68.2)	4230 (75.8)
Safe		1865 (32.9)	1400 (25.3)	1135 (20.3)
Neither safe nor unsafe		606 (10.7)	299 (5.4)	169 (3.0)
Unsafe		237 (4.2)	50 (0.9)	37 (0.7)
Very unsafe		45 (0.8)	12 (0.2)	10 (0.2)
**Relational factors**				
*Bullying (past year)*	1.0			
Never		4552 (79.9)	4887 (88.2)	4461 (80.1)
2–3 times a month		660 (11.6)	414 (7.5)	635 (11.4)
Weekly		164 (2.9)	106 (1.9)	166 (3.0)
Most days		254 (4.5)	97 (1.8)	237 (4.3)
Every day		70 (1.2)	37 (0.7)	71 (1.3)
*Bullying (change during lockdown) for those bullied in the past year*	1.7			
Much less		674 (59.7)	414 (64.6)	878 (80.4)
Slightly less		236 (20.9)	120 (18.7)	131 (12.0)
The same amount		79 (7.0)	50 (7.8)	25 (2.3)
Slightly more		91 (8.1)	42 (6.6)	40 (3.7)
Much more		49 (4.3)	15 (2.3)	18 (1.6)
*Friend relationships (reference)*	1.5			
Very well or well		3622 (63.8)	4068 (73.8)	4006 (72.6)
Most of the time well		1861 (32.8)	1394 (25.3)	1422 (25.8)
Not well or not at all well		190 (3.3)	52 (0.9)	90 (1.6)
*Friend relationships (change during lockdown)*	7.7			
Much better		319 (5.9)	251 (4.7)	674 (12.9)
Better		1121 (20.9)	1149 (21.7)	1468 (28.1)
The same		2669 (49.8)	3334 (62.9)	2433 (46.6)
Less		996 (18.6)	500 (9.4)	543 (10.4)
Much less		258 (4.8)	70 (1.3)	106 (2.0)
*Family relationships (reference)*	0.9			
Very well or well		1741 (30.5)	2723 (49.1)	3158 (56.7)
Most of the time well		3423 (60.0)	2701 (48.7)	2299 (41.3)
Not well or not at all well		540 (9.5)	120 (2.2)	112 (2.0)
*Family relationships (change during lockdown)*	6.4			
Much better		178 (3.3)	212 (4.0)	825 (15.5)
Better		988 (18.1)	1178 (22.1)	1969 (37.0)
The same		2271 (41.7)	3220 (60.4)	2039 (38.3)
Less		1652 (30.3)	665 (12.5)	445 (8.4)
Much less		361 (6.6)	57 (1.1)	47 (0.9)
*Feeling left out (reference)*	1.8			
Never		1609 (28.4)	3125 (56.8)	3042 (55.1)
Sometimes		2881 (50.9)	2048 (37.2)	2087 (37.8)
Often		1167 (20.6)	329 (6.0)	390 (7.1)
*Feeling left out (change during lockdown)*	9.3			
Much less		486 (9.1)	567 (10.9)	1273 (24.7)
Slightly less		1193 (22.4)	1066 (20.6)	1327 (25.8)
The same amount		2062 (38.7)	2948 (56.9)	1884 (36.6)
Slightly more		1257 (23.6)	542 (10.5)	564 (10.9)
Much more		325 (6.1)	57 (1.1)	103 (2.0)
*Loneliness (reference)*	1.6			
Never		1295 (22.8)	3349 (61.0)	3391 (61.3)
Sometimes		2680 (47.2)	1750 (31.9)	1784 (32.2)
Often		1702 (30.0)	393 (7.2)	360 (6.5)
*Loneliness (change during lockdown)*	8.0			
Much less		315 (5.7)	485 (9.3)	1226 (23.7)
Slightly less		643 (11.7)	734 (14.1)	1082 (21.0)
The same amount		1342 (24.4)	2874 (55.2)	1789 (34.7)
Slightly more		2315 (42.2)	974 (18.7)	936 (18.1)
Much more		877 (16.0)	143 (2.7)	130 (2.5)
Concern about appearance*	2.6			
Not at all worried		276 (7.3)	605 (19.5)	631 (20.1)
Not very worried		641 (16.9)	984 (31.7)	904 (28.8)
Quite worried		645 (17.0)	671 (21.6)	605 (19.2)
Worried		1163 (30.7)	574 (18.5)	705 (22.4)
Extremely worried		1069 (28.2)	275 (8.8)	298 (9.5)
**Lifestyle factors**				
*Exercise (left house during lockdown)*	5.1			
Every day		985 (18.0)	1062 (19.8)	1238 (23.1)
Most days		2214 (40.5)	2459 (45.8)	2454 (45.9)
Sometimes		1218 (22.3)	1177 (21.9)	931 (17.4)
Once or twice		758 (13.9)	495 (9.2)	511 (9.5)
Not at all		287 (5.3)	178 (3.3)	217 (4.1)
*Exercise (change during lockdown)*	4.5			
Much more		496 (9.0)	475 (8.8)	861 (16.0)
Slightly more		1126 (20.4)	1197 (22.2)	1514 (28.2)
The same amount		533 (9.6)	964 (17.9)	721 (13.4)
Slightly less		2091 (37.8)	2106 (39.1)	1711 (31.8)
Much less		1281 (23.2)	650 (12.1)	567 (10.6)
*Sleep (change during lockdown)*	9.6			
Much more		224 (4.1)	332 (6.6)	869 (17.1)
Slightly more		816 (15.1)	1172 (23.3)	1600 (31.5)
The same amount		1005 (18.6)	2012 (40.0)	1387 (27.3)
Slightly less		2316 (42.8)	1292 (25.7)	1022 (20.1)
Much less		1052 (19.4)	223 (4.4)	205 (4.0)

*Not asked of primary school pupils

#### Sociodemographic characteristics

Supplementary Fig. 2 shows responses by gender, key stage, free school meal eligibility, immigration status, and garden access. Sociodemographic characteristics were similar in the groups who reported improvement in their mental wellbeing and those who reported no change, whilst the group who reported deterioration in their wellbeing had proportionally more girls and older students.

#### School factors

In terms of school attendance, the highest proportions of students who reported improved mental wellbeing were amongst those who were in school every day (39.2%), most days (34.7%), or not at all (33.5%) (Fig. 1). For the four reference variables studied (i.e. perceptions of safety at school, concern about school performance, and academic support at school and at home), those who reported improved mental wellbeing were largely similar to those who reported no change, and each of these groups reported more positive experiences than did the group who reported deterioration (Table 1).

**Fig. 2:**
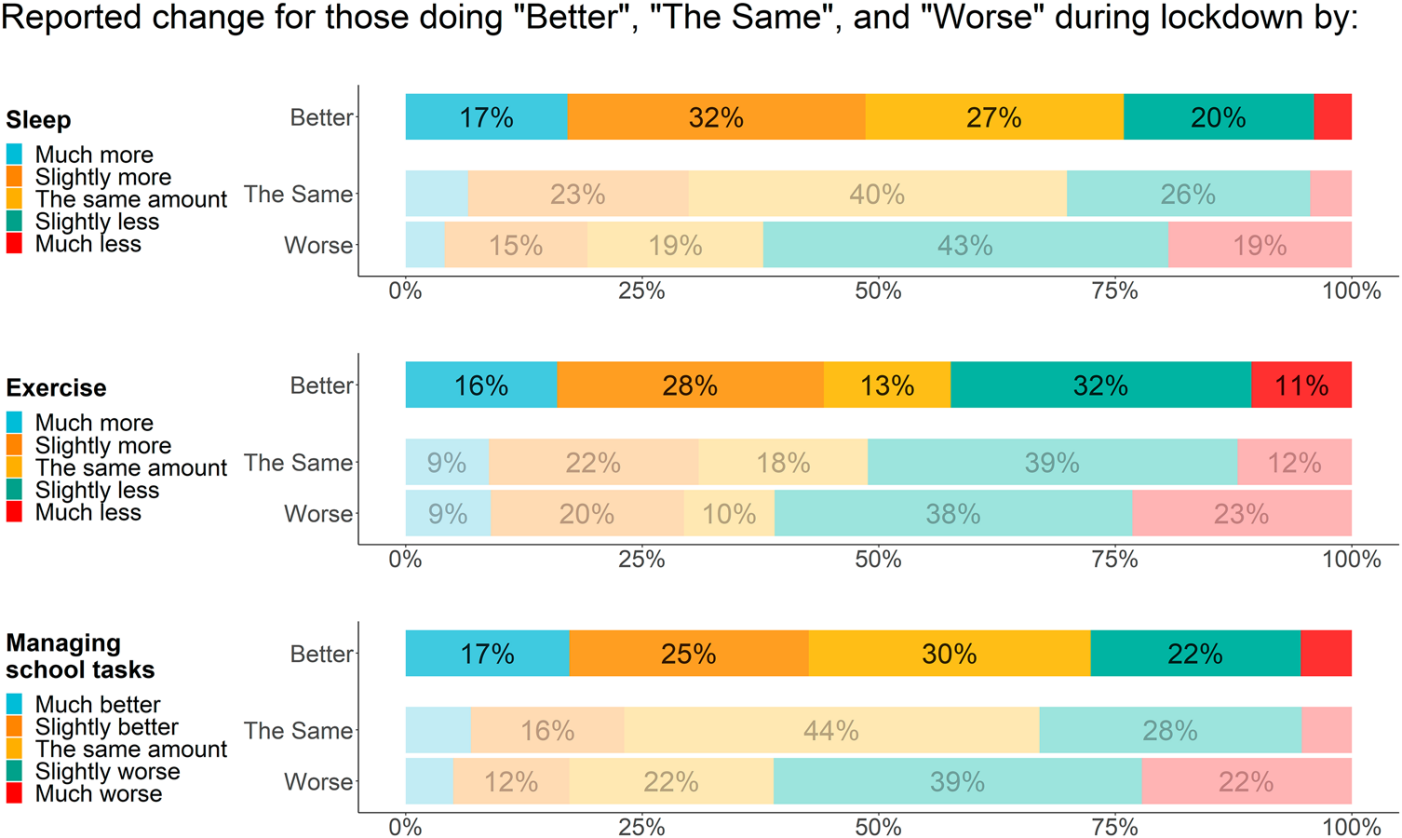
Self-reported changes in mental wellbeing and lifestyle and school factors

In terms of self-reported changes during lockdown, the proportion of students who reported that they were managing their school tasks ‘slightly’ or ‘much’ better than before lockdown was higher for the group who reported improved mental wellbeing (42.6%) than for the groups who reported no change (23.1%) or deterioration (17.3%) (Fig. 2).

#### Home factors

The proportion of students who reported that they felt ‘safe’ or ‘very safe’ at home was similar for those who reported improvement (96.1%) and no change (93.5%) in mental wellbeing and lower for those who reported deterioration (84.3%).

#### Relational factors

For the six reference variables studied (i.e. past year experience of bullying, family relationships, friend relationships, feeling lonely, feeling left out, and concern about appearance), those who reported improved mental wellbeing were largely similar to those who reported no change, and each of these groups reported more positive experiences than did the group who reported deterioration (Table 1).

**Fig. 3:**
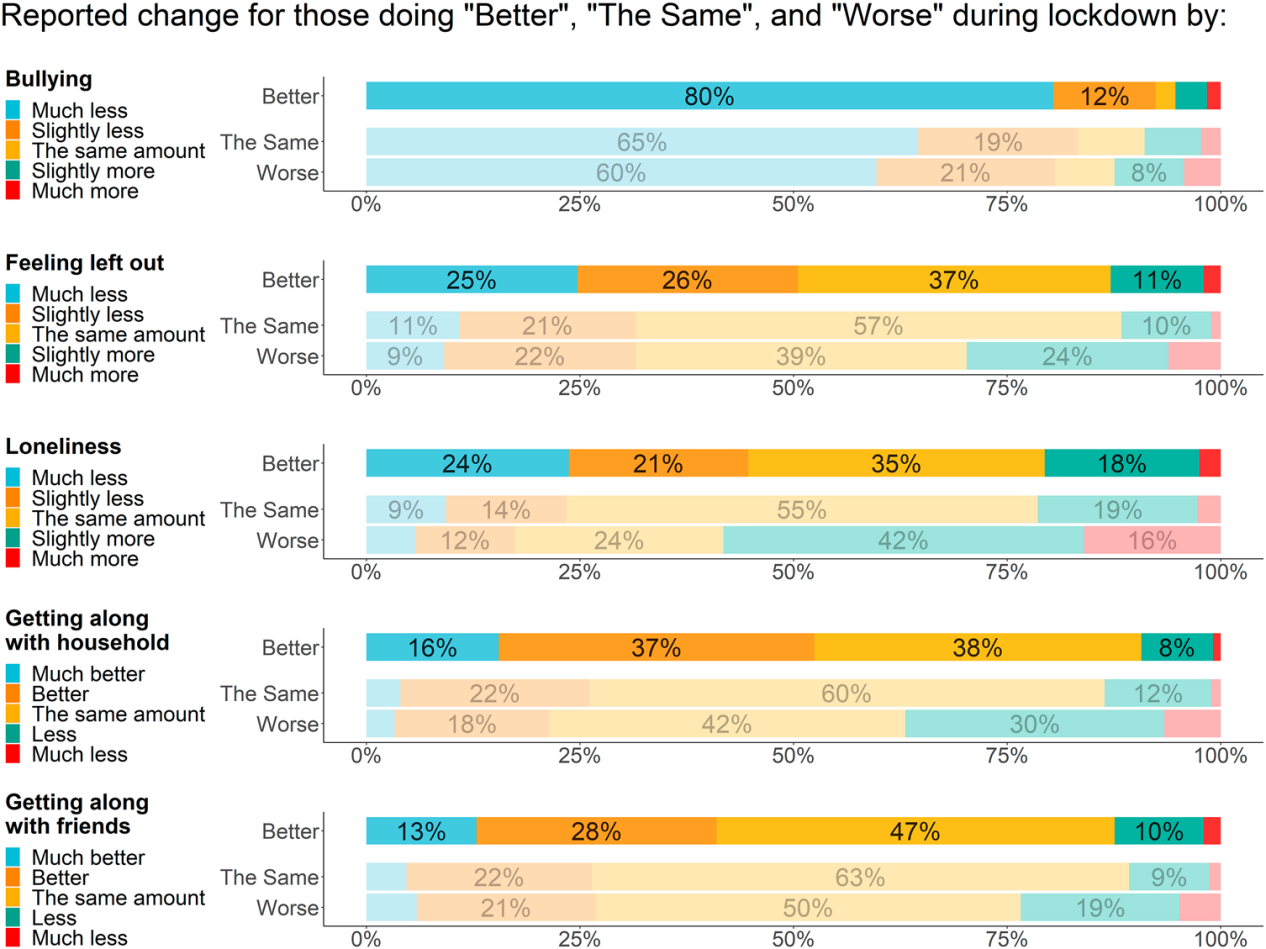
Self-reported changes in mental wellbeing and relational factors

In terms of self-reported changes during lockdown, a higher proportion of those who reported improved mental wellbeing also reported improvements across all relational factors studied (Fig. 3). The proportion of students who reported that they were getting along with household members ‘better’ or ‘much better’ than before lockdown was higher for the group who reported improved mental wellbeing (52.5%) than for the groups who reported no change (26.1%) or deterioration (21.4%), with a similar pattern for getting along with friends (41.0%, 26.4%, and 26.8% of those who reported improvement, no change, or deterioration, respectively). Feeling less left out or lonely during lockdown was also more common in this group: of those who reported improved mental wellbeing, 44.7% said they were ‘slightly’ or ‘much’ less lonely than before lockdown, compared with 23.4% and 17.4% of those who reported no change and deterioration, respectively. Furthermore, 50.5% of those who reported improved mental wellbeing reported feeling ‘slightly’ or ‘much’ less left out, compared with 31.5% of those who reported no change and 31.5% of those who reported deterioration. Finally, whilst all groups reported reduced bullying during lockdown, the proportion that reported that they were bullied ‘slightly’ or ‘much’ less than before lockdown was higher for those who reported improved wellbeing (92.4%) than for those who reported no change (83.3%) or deterioration (80.6%).

#### Lifestyle factors

The proportion of students who reported that they left their house to exercise ‘most days’ or ‘every day’ was similar for those who reported improvement (69.0%) or no change (65.6%) in mental wellbeing and lower for those who reported deterioration (58.5%). In terms of self-reported changes during lockdown, the proportion of students who reported that they were exercising ‘much’ or ‘slightly’ more than before lockdown was higher for the group who reported improved mental wellbeing (44.2%) than for the groups who reported no change (31.0%) or deterioration (29.4%). Similarly, 48.6% of those who reported improved mental wellbeing reported sleeping ‘slightly’ or ‘much’ more, compared with 29.9% of those who reported no change and 19.2% of those who reported deterioration.

#### Feelings about returning to school after lockdown

**Fig. 4:**
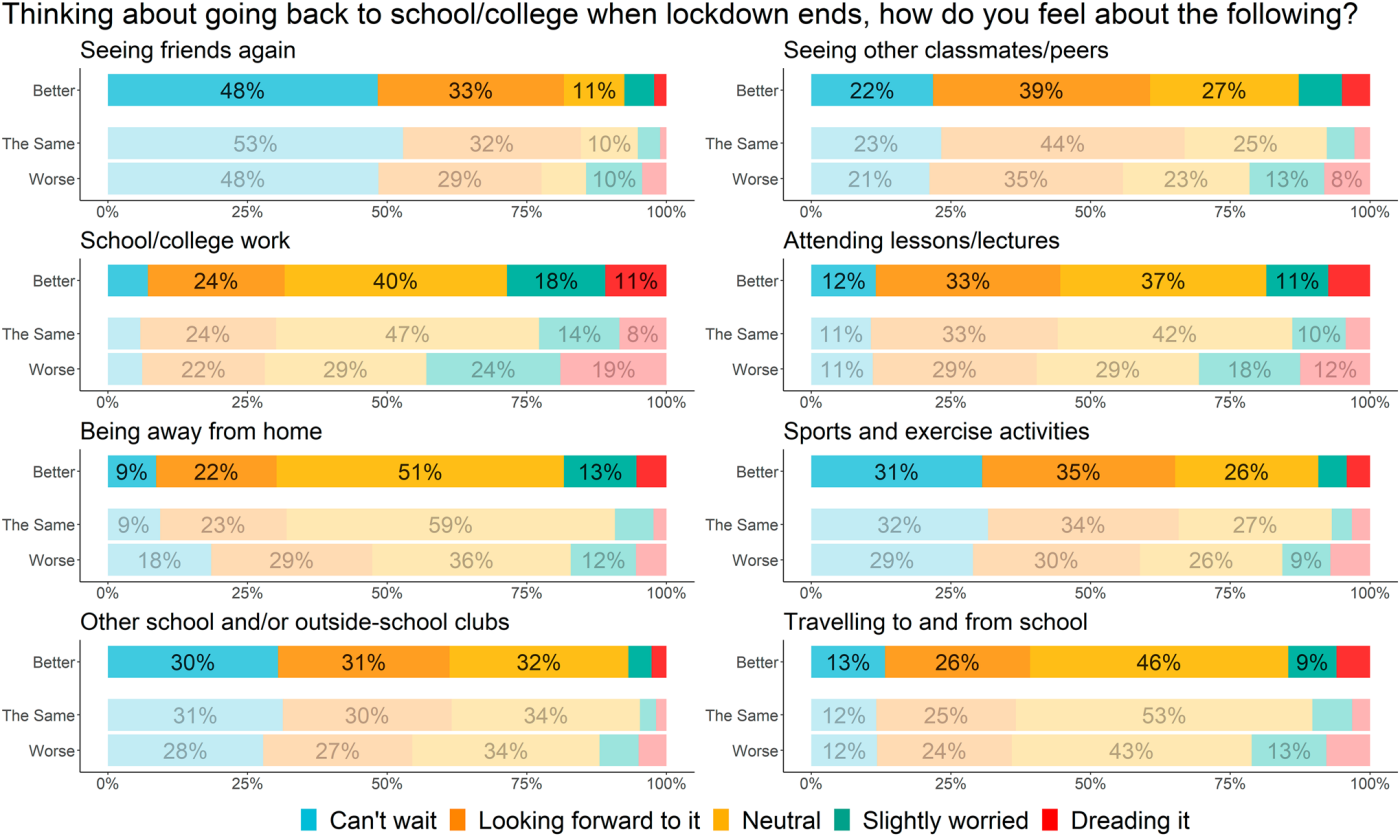
Self-reported changes in mental wellbeing and feelings about returning to school

Differences in terms of feelings about returning to school were generally not as large as for other questions (Fig. 4; Supplementary Table 3). Excluding concerns about being away from home, the trend across all eight aspects was that the proportion ‘slightly worried’ about or ‘dreading’ the different aspects of school return was highest for those who reported deterioration in mental wellbeing, followed by those who reported improvement, and finally those who reported no change. Whilst students were generally positive or neutral about school return, aspects that the greatest number of students were concerned about were schoolwork (43.0% of students who reported deterioration, 28.6% of those who reported improvement, and 22.8% of those who reported no change), attending lessons (30.6%, 18.6%, and 13.9%, respectively), being away from home (17.2%, 18.4%, and 9.3%, respectively), and travelling to and from school (21.2%, 14.6%, and 10.3%, respectively).

## Discussion

This large school-based study showed that one-third of students reported an improvement in their mental wellbeing during the first UK Covid-19 lockdown, representing a similar proportion to those who reported no change or deterioration to their wellbeing. For many of the reference variables studied (i.e. those that asked about general experiences versus lockdown-specific changes), students who reported improvement were quite similar to their peers who reported no change, and both groups fared better than those who reported deterioration. However, for the variables that measured self-reported *changes during lockdown*, CYP who reported improved mental wellbeing were more likely than their peers to report improvement across the full range of school, relational, and lifestyle factors studied.

This study adds to a growing evidence base that suggests that the impact of lockdown is dependent on a number of factors (e.g. gender, pre-pandemic mental health, social relationships, school connectedness, experience of online learning, family composition, and family financial situation [4, 5, 7–9, 11]) and that there are many CYP who report experiencing better mental health and wellbeing during this time [8, 9, 11]. Whilst our estimate that one in three CYP experienced improved mental wellbeing during lockdown is higher than estimates from other studies, it is difficult to directly compare due to different sampling techniques and outcome measures, as well as variability in the nature of and response to the pandemic across geographical and chronological contexts. One of the most similar studies in terms of pandemic context is the English National Survey [1], which was conducted during the same timeframe as our survey. The estimate from this study, that 27% of 11–16-year-olds said that lockdown had made their lives better, is fairly comparable to our own. A Canadian study conducted around the same time (during ‘national emergency measures’ including school closures) had a slightly lower estimate, with around 20% of CYP experiencing an improvement in at least one domain of mental health.

The patterns in changes in mental wellbeing by school attendance during lockdown are interesting. Various aspects of the school environment, including bullying [28, 29], academic stress [30–32], and poor relationships with teachers [33], are linked to poor mental health. It would, therefore, be reasonable to expect that being away from the school environment could be beneficial for certain students [18]. However, we found that those attending school ‘most days’ or ‘every day’ (though they made up a small percentage of the total sample) had a higher proportion of students who reported improved mental wellbeing compared with those attending irregularly. Interpreting these findings requires nuance, however, and our findings cannot determine whether school attendance was driving changes in mental wellbeing. As described in the Methods section, at the time of the survey, only certain students were eligible for in-school provision. Additionally, an analysis of secondary school students from the OxWell Survey demonstrated that contextual and background factors, such as previous access to mental health support, may explain the relationship between school attendance and change in mental wellbeing [19]. For those in school, the experience was likely substantially different, influenced by the demographic and environmental school context. Some would have had their same teachers in smaller class sizes whilst others might have experienced blended lessons with other students whom they might never have learned with before. Some would have had none of their friendship group with them at school, whilst others might have been able to be with friends and/or away from more difficult interpersonal relationships. Discussions with stakeholders (including CYP, parents, and teachers) suggest that this altered provision may well have been a positive experience, with potential for greater attention from teachers, more tailored learning, and increased focus on wellbeing.

This study also highlights the importance of relationships. The potential for increased social isolation during the pandemic is concerning, particularly for young people, for whom peer interactions are especially important [34, 35]. Widnall and colleagues [8] reported during the first UK lockdown that 42% of girls and 27% of boys worried about the impact of lockdown on friendships. Concerns about friendships were also common in young people's qualitative accounts of the pandemic, with many citing disruptions to their social networks and feelings of loneliness and isolation [12, 13, 15, 16]. Yet, nearly half of those who reported improved mental wellbeing in our sample reported feeling less left out and lonely and having better relationships with friends and family. There are a number of reasons why this may be. As Orben and colleagues [34] have suggested, most CYP have access to digital forms of social interaction that can mitigate the negative effects of reduced face-to-face contact. This is also reflected in Widnall and colleagues’ [8] finding that peer connectedness remained largely stable during the pandemic. With many parents and carers at home, there was also potential for improved family relationships, a fact empirically demonstrated in Penner and colleagues’ [9] finding that better family functioning was associated with better mental health during the first period of ‘stay at home’ orders and school closures in the United States.

One specific aspect of peer relationships that changed during the pandemic was bullying. To our knowledge, this is the first study to quantify the relationship between change in bullying and change in mental wellbeing during lockdown. At the start of the pandemic, some experts were worried that bullying, and cyberbullying in particular, could increase [32, 36]. Others believed that CYP who experience bullying at school would fare better during the lockdown due to increased control over social interactions and greater ability to focus on academic study [18]. In terms of empirical evidence, Silk and colleagues [15] found that around half of the nearly 100 adolescent girls in their diary-based study listed the ability to avoid unwanted interactions with classmates as a positive impact of the pandemic. We found that most CYP who had been bullied in the past year reported that the bullying had reduced, and given the well-established links between bullying and mental health [37], it was unsurprising that reductions were most prevalent amongst those who reported improved mental wellbeing.

For approximately half of the CYP who reported improved mental wellbeing, lockdown was associated with improvements in sleep and exercise. Sleep is crucial for CYP’s mental health and wellbeing [38], and it was not initially clear how the pandemic would affect sleep [32]. In their study of sleep during Singapore’s national lockdown in April–June 2020 (which included school closures), Lim and colleagues [39] found that, on average, CYP got more sleep during this time, and Silk and colleagues [15] found that 87% of the girls in their study reported increased sleep as a positive impact of the pandemic. These findings support discussions as to whether typical school start times are optimal for CYP’s sleep. In examining the role of exercise, a more complicated picture emerged. Whilst CYP were encouraged to continue exercising during the pandemic, lockdown restrictions often limited opportunities for outdoor and group exercise. Interestingly, in Silk and colleagues’ [15] diary study, three-quarters of the girls reported having more time to exercise or go outdoors. In our survey, whilst more CYP who reported improved mental wellbeing reported doing more exercise, there was a bimodal distribution, with as many reporting doing more exercise as doing less.

This study also highlighted the aspects of school return that students most looked forward to and those that they worried about most. The most anticipated aspect of school return was seeing friends again, whilst other positive aspects included seeing classmates and peers and participating in sports and other activities, all of which highlight the central role of peer interaction in CYP’s lives [34]. This finding resonates with a key theme from Fisher and colleagues’ [12] qualitative interviews, namely that the majority of young people were looking forward to going back to school and seeing their friends after the first UK lockdown. Conversely, the aspects causing the most concern were primarily related to school systems, including lessons and schoolwork. This reflects findings from other studies that school and academic concerns including disrupted learning and uncertainty about upcoming examinations were common amongst CYP during lockdown [8, 12, 15, 16]. For some CYP, lockdown will have meant a reduction in academic stress or a more flexible learning system that better suited their needs, each of which may contribute to improved mental health and wellbeing.

### Limitations

We acknowledge six main limitations. First, whilst our sample is large and diverse, it may not be representative of the general UK population, and we did not conduct a weighted analysis to account for potential differences between our sample and the population. For example, participants were from the south of England, which is relatively affluent compared with other areas of the country. Additionally, most students had to complete the survey from home, which could exclude students without the necessary resources (a computer, internet) or other vulnerable students (e.g. those who do not have a safe home environment). Second, due to the repeated cross-sectional design of the survey, we do not have (individually linkable) pre-pandemic data to indicate objective change in mental wellbeing or in any of our other variables of interest. Third, our validated measure of wellbeing (the WEMWBS) was slightly modified in the OxWell survey to be answered on a sliding scale, which potentially could have altered its psychometric properties. Fourth, there are several relevant variables missing from our data. Due to the ethical requirements associated with obtaining opt-out consent, we were not able to collect information on students’ ethnicity or include gender options beyond male/female. In the stakeholder questionnaire, parents and CYP also identified a number of potentially relevant variables that were not available in the OxWell survey data, including factors related to school systems (e.g. flexible learning, autonomy over school work, class sizes) and neurodiversity (e.g. autism, sensory needs). These are key variables that warrant further study, ideally using mixed methods that include qualitative interviews. Fifth, we collected data only during the first lockdown; anecdotal experience from CYP, families, and school staff members indicates that this lockdown was qualitatively different from the others, but we were not able to explore this. Finally, we primarily aimed to explore the characteristics of those reporting better wellbeing during lockdown and describe their feelings and behaviours rather than model the relative contribution of these factors to their reported wellbeing. There are likely to be complex inter-relationships between the factors we measured and whether a pupil had improved wellbeing during lockdown, which would benefit from further exploration.

### Lessons learned and consequences for the future

This study provides insight into individual and environmental factors that may support CYP to thrive in the context of school disruption and adversity. First, considering educational differences, there were increased opportunities during lockdown for flexible and tailored teaching that encouraged different styles of learning and student autonomy over schedule and schoolwork [12, 14, 15, 18]. For those in school, smaller class sizes and more focused attention from teachers might have had a positive impact to wellbeing, whilst later wake times [32, 39] and more freedom during the school day [15, 18] might have benefitted students at home. For some, there was more focus on sport, play, and the creative arts [12, 14, 15], which may have contributed to wellbeing, although it is likely that this was more accessible for only a minority of students. For certain students, there may also have been fewer ‘typical school day’ distractions (e.g. negative comparisons with other students, school-based anxiety, sensory challenges, concerns about disciplinary action, and uniform requirements [8, 18]). Finally, there was a greater emphasis in both schools and wider society on maintaining wellbeing [40, 41].

Second, interpersonal relationships during the pandemic were altered. Some CYP experienced improvements in relationships with family and friends [9, 14–16], often facilitated by social media and other digital platforms [8, 12, 16, 42]. CYP who were in school during this time may have had the opportunity to make new friendships, whilst others experienced a respite from negative relationships [15, 18, 19]. All groups in this study reported less bullying during lockdown, potentially reflecting better opportunities to control their exposures to peers who might have been targeting them in the classroom or during unstructured times in the school day (e.g. on the journey to/from school or in break times)﻿.

Taken together, these findings challenge the dominant narrative that the pandemic and accompanying lockdown measures have had overwhelmingly adverse effects on the lives of CYP. Determining the reasons *why* some CYP felt they fared better during lockdown and considering *how* these beneficial experiences can be maintained beyond the pandemic might provide valuable insights into mental health and wellbeing not only for these students, but for the millions of young people being educated during the Covid-19 pandemic. Based on our findings, a dual emphasis at school on both achievement and building interpersonal relationships seems essential. Furthermore, additional flexibility on certain aspects of school structure and expectations might better support CYP’s mental health and wellbeing. Importantly, the responsibility to conceptualise and implement any needed changes cannot fall only on individual schools or educators, but instead must be part of a systemic shift including all those working with school-aged CYP and their families.

In summary, CYP have had diverse experiences of the pandemic, influenced by a multitude of interacting individual-, family-, and community-level risk and protective factors. The negative impacts to the lives of many affected by illness, socioeconomic stressors, and disruptions to family and community life cannot be ignored. However, some CYP have experienced positive changes during the pandemic, which necessitate further examination, exploration, and reflection.

## Supplementary Information

Below is the link to the electronic supplementary material.

Supplementary file1 (PNG 835 KB)

Supplementary file2 (PNG 145 KB)

Supplementary file3 (DOCX 19 KB)

Supplementary file4 (DOCX 17 KB)

Supplementary file5 (DOCX 17 KB)

## Data Availability

Fully anonymous extracts of the data can be provided to academic research collaborators upon reasonable request, following a careful review process by the research team. The data are not publicly available due to ethical and information governance restrictions.

## Code availability

The code used in this analysis is available on request from the authors.
