# Supplementary figures and images for: Happier during lockdown: a descriptive analysis of self-reported wellbeing in 17,000 UK school students during Covid-19 lockdown
Source: Eur Child Adolesc Psychiatry. 2022 Feb 17;32(6):1131–46. doi: 10.1007/s00787-021-01934-z (PMC8853175; doi:10.1007/s00787-021-01934-z)

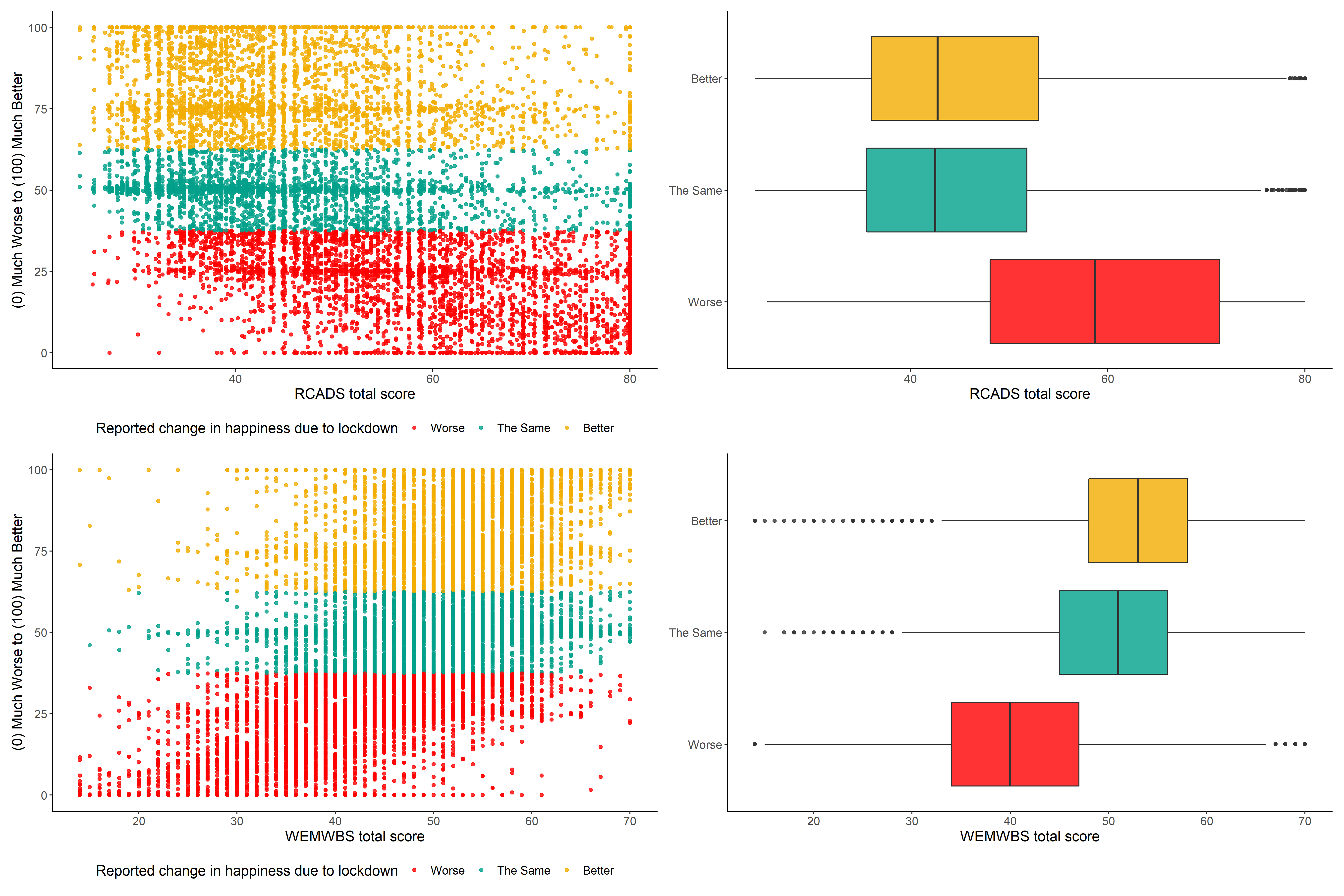

Supplement: Supplementary file 1 — Supplementary file1 (PNG 835 KB) [file 787_2021_1934_MOESM1_ESM.png]

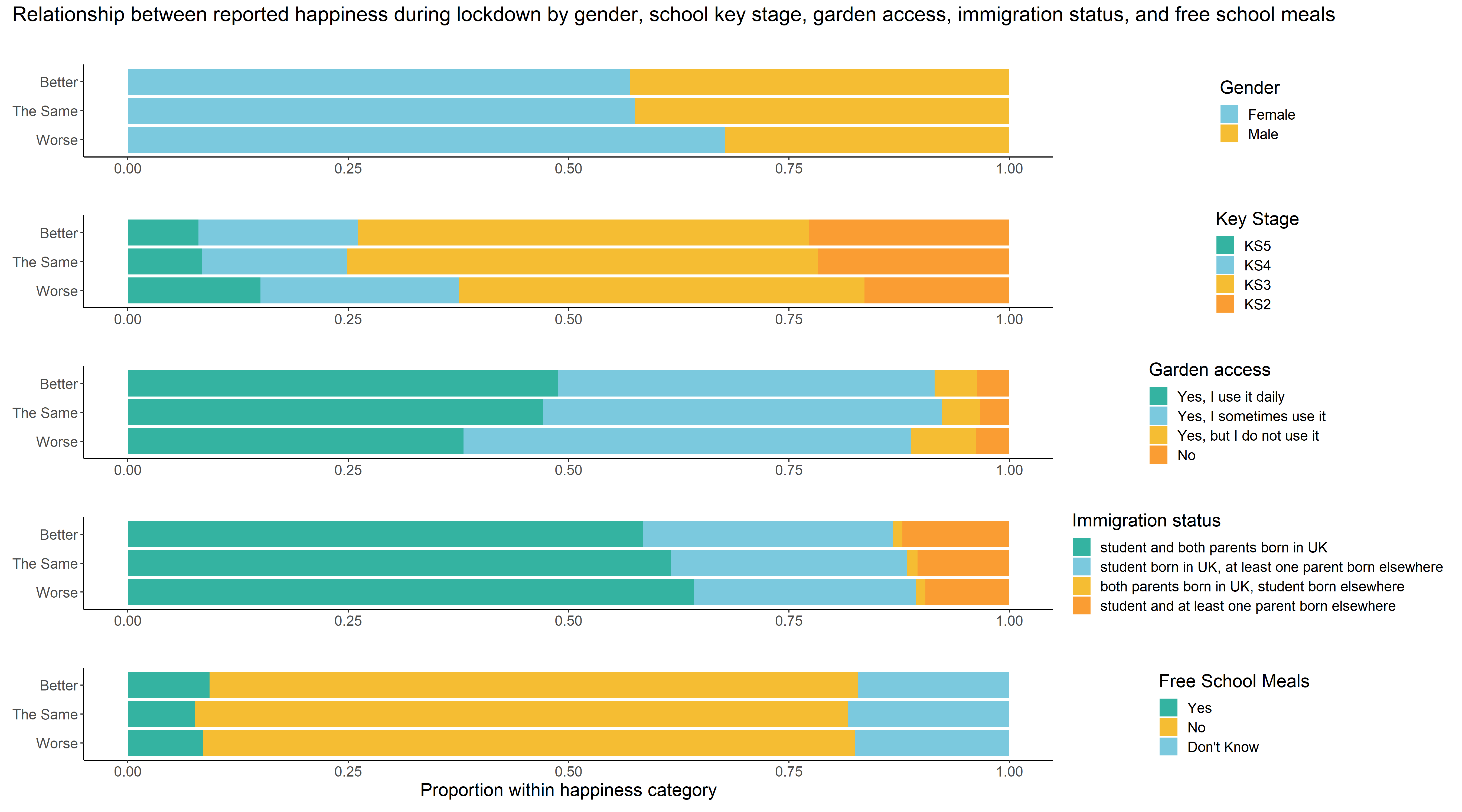

Supplement: Supplementary file 2 — Supplementary file2 (PNG 145 KB) [file 787_2021_1934_MOESM2_ESM.png]
