# Supplementary material for: Happier during lockdown: a descriptive analysis of self-reported wellbeing in 17,000 UK school students during Covid-19 lockdown
Source: Eur Child Adolesc Psychiatry. 2022 Feb 17;32(6):1131–46. doi: 10.1007/s00787-021-01934-z (PMC8853175; doi:10.1007/s00787-021-01934-z)
Supplement: Supplementary file 3 — Supplementary file3 (DOCX 19 KB) [file 787_2021_1934_MOESM3_ESM.docx]

**Supplementary Table 1.** Stakeholder suggestions and their closest matching OxWell Survey variables

| **Category** | **Suggestions from stakeholder survey** | **Related variable in OxWell data** |
| --- | --- | --- |
| **School systems** | 1:1 learning/more support with learning | - Academic support at home - Academic support at school |
|  | Can learn at own pace | - School task management (change during lockdown) |
|  | Fewer interruptions/ distractions to learning | - School task management (change during lockdown) - Attending lessons (feelings about school return) |
|  | Flexible/remote learning | - Academic support at home - Academic support at school - School task management (change during lockdown) |
|  | Less disciplinary action | *None* |
|  | Less stress/pressure | - School task management (change during lockdown) - Concern about school performance - School work (feelings about school return) |
|  | Less work/no exams | - School task management (change during lockdown) - Concern about school performance - School work (feelings about school return) |
|  | More autonomy/control over schedule and schoolwork | - School task management (change during lockdown) - School work (feelings about school return) |
|  | More focus on wellbeing | *None* |
|  | More freedom/free time | *None* |
|  | No school | - School attendance - Attending lessons (feelings about school return) |
|  | No uniform | *None* |
|  | Smaller class sizes | - Academic support at school |
| **Home** | Alone time | *None* |
|  | Could stay at home | - School attendance - Being away from home (feelings about school return) |
|  | Home is a safe space | - School attendance - Being away from home (feelings about school return) - Safety at home |
|  | Home is happier | - School attendance - Being away from home (feelings about school return) |
|  | More relaxed environment | - Being away from home (feelings about school return) |
|  | Needs are met at home | - Being away from home (feelings about school return) - Academic support at home |
| **Relational changes** | Away from crowds | *None* |
|  | Away from peers | - Seeing other classmates/peers (feelings about school return) |
|  | Away from professionals | *None* |
|  | Away from teachers | - Attending lessons (feelings about school return) |
|  | Fewer friendship issues | - Friend relationships (reference & change during lockdown) - Seeing friends again (feelings about school return) |
|  | Fewer social obligations/expectations | - Seeing other classmates/peers (feelings about school return) - Other school and/or outside school clubs (feelings about school return) |
|  | Less competition | - Concern about academic performance - Seeing other classmates/peers (feelings about school return) - Sports and exercise activities (feelings about school return) - Other school and/or outside school clubs (feelings about school return) |
|  | Less peer pressure | - Seeing other classmates/peers (feelings about school return) |
|  | Less pressure about appearance | - Concern about appearance |
|  | Less social pressure | - Seeing other classmates/peers (feelings about school return) - Seeing friends again (feelings about school return) |
|  | More time with family | - Family relationships (reference & change during lockdown) - Being away from home (feelings about school return) |
|  | More time with pets | *None* |
|  | No bullying | - Bullying (past year & change during lockdown) - Safety at school - Seeing other classmates/peers (feelings about school return) - Feeling left out (reference & change during lockdown) |
|  | Remote friendships/ interactions | - Friend relationships (reference & change during lockdown) - Seeing friends again (feelings about school return) |
| **Mental health/ SEN/neurodiversity** | Fewer sensory issues | *None* |
|  | Improved attachment | *None* |
|  | Improved confidence/self-esteem | - Wellbeing (Warwick Edinburgh Mental Wellbeing Scale) - Concern about appearance |
|  | Less anxiety (general, social, separation) | - Anxiety (Revised Child Anxiety and Depression Scale) |
|  | No masking necessary | *None* |
| **Miscellaneous** | Calmer daily/morning routines | - Travelling to and from school (feelings about school return) - Sleep (change during lockdown) |
|  | Calmness/quiet time | *None* |
|  | Everyone is in the same situation | - Feeling left out (reference & change during lockdown) - Feeling lonely (reference & change during lockdown) |
|  | Freedom to move around/fidget/sing/etc. | *None* |
|  | Improved health (e.g. eating, skin) | *None* |
|  | Learning new skills | *None* |
|  | Less time pressure | *None* |
|  | Love & support | *None* |
|  | More play | *None* |
|  | No travel to school | - Travelling to and from school (feelings about school return) |
|  | Physical activity | - Exercise (left house during lockdown & change during lockdown) - Sports and exercise activities (feelings about school return) |
|  | Sleep (more/better/later) | - Sleep (change during lockdown) |
|  | Slower pace of life | *None* |
|  | Tech access (phone) | *None* |
|  | Time for hobbies/interests | *None* |
|  | Time outdoors | - Exercise (left house during lockdown) - Garden access |
