# Supplementary material for: Happier during lockdown: a descriptive analysis of self-reported wellbeing in 17,000 UK school students during Covid-19 lockdown
Source: Eur Child Adolesc Psychiatry. 2022 Feb 17;32(6):1131–46. doi: 10.1007/s00787-021-01934-z (PMC8853175; doi:10.1007/s00787-021-01934-z)
Supplement: Supplementary file 4 — Supplementary file4 (DOCX 17 KB) [file 787_2021_1934_MOESM4_ESM.docx]

**Supplementary Table 2.** Selected variables, question wordings, and response options

| **Variable** | **Question** | **Response options*** |
| --- | --- | --- |
| **Sociodemographic factors** | | |
| Gender | Are you a boy or a girl? | Girl – boy |
| Year group | Please enter your Year Group | 4 – 5 – 6 – 7 – 8 – 9 – 10 – 11 – 12 – 13 |
| Free school meal eligibility | Do you have (i.e. are you eligible for) free school lunches? | Yes – No – Don't know |
| Immigration status | Were you born in the UK?  Were both of your parents born in the UK? | Yes – No – Would rather not say  Yes – No – Would rather not say |
| Garden access | Do you have access to a garden? | No – Yes, but I don't use it – Yes, I sometimes use it – Yes, I use it every day |
| **School factors** | | |
| School attendance | During lockdown, have you left the house to go to school/college? | Sliding scale 0-100 with following labels: Not at all (0-12.5) – Once or twice (12.6-37.5) – Sometimes (37.6-62.5) – Most days (62.6-87.5) – Every day (87.6-100) |
| Safety at school | When you are at school, how safe do you feel? | Sliding scale 0-100 with following labels: Very unsafe (0-12.5) – Unsafe (12.6-37.5) – Neither safe nor unsafe (37.6-62.5) – Safe (62.6-87.5) – Very safe (87.6-100) |
| Concern about school performance** | *Some young people feel worried about things happening around them. To what extent do you worry about the following topics:*  Doing well at school | Sliding scale 0-100 with following labels: Not at all worried (0-12.5) – Not very worried (12.6-37.5) – Quite worried (37.6-62.5) – Worried (62.6-87.5) – Extremely worried (87.6-100) |
| Academic support at home | How much help and support do you feel you get with your homework from the people you live with? | Sliding scale 0-100 with following labels: No help at all (0-12.5) – Not enough help (12.6-37.5) – Just about enough help (37.6-62.5) – Most of the help I need (62.6-87.5) – All of the help I need (87.6-100) |
| Academic support at school | How much help and support with learning do you feel that you get at school? | Sliding scale 0-100 with following labels: No help at all (0-12.5) – Not enough help (12.6-37.5) – Just about enough help (37.6-62.5) – Most of the help I need (62.6-87.5) – All of the help I need (87.6-100) |
| School task management (change during lockdown) | During lockdown, how have you been managing your school tasks? | Sliding scale 0-100 with following labels: Much worse (0-12.5) – Slightly worse (12.6-37.5) – The same (37.6-62.5) – Slightly better (62.6-87.5) – Much better (87.6-100) |
| **Home factors** | | |
| Safety at home | How safe do you feel at home or the place where you live? | Sliding scale 0-100 with following labels: Very unsafe (0-12.5) – Unsafe (12.6-37.5) – Neither safe nor unsafe (37.6-62.5) – Safe (62.6-87.5) – Very safe (87.6-100) |
| **Relational factors** | | |
| Bullying (past year) | Have you been bullied in the last year (at least 2-3 times a month)? | Sliding scale 0-100 with following labels: Never or just teased a few times (0-12.5) – 2-3 times a month (12.6-37.5) – Weekly (37.6-62.5) – Most days (62.6-87.5) – Every day (87.6-100) |
| Bullying (change during lockdown) *for those bullied in the past year* | During lockdown, has the bullying happened less, the same or more than before? | Sliding scale 0-100 with following labels: Much less (0-12.5) – Slightly less (12.6-37.5) – The same amount (37.6-62.5) – Slightly more (62.6-87.5) – Much more (87.6-100) |
| Friend relationships (reference) | How well do you get along with your friends? | Very well or well – Most of the time well – Not well or not at all well |
| Friend relationships (change during lockdown) | During lockdown, have you got along less well, the same or better with your friends? | Sliding scale 0-100 with following labels: Much less (0-12.5) – Slightly less (12.6-37.5) – The same (37.6-62.5) – Better (62.6-87.5) – Much better (87.6-100) |
| Family relationships (reference) | How well do you get along with other people in your household? | Very well or well – Most of the time well – Not well or not at all well |
| Family relationships (change during lockdown) | During lockdown, have you got along less well, the same or better with other people in your household? | Sliding scale 0-100 with following labels: Much less (0-12.5) – Slightly less (12.6-37.5) – The same (37.6-62.5) – Better (62.6-87.5) – Much better (87.6-100) |
| Feeling left out (reference) | How often do you feel left out? | Hardly ever or never – Some of the time – Often |
| Feeling left out (change during lockdown) | During lockdown, have you felt less, the same or more left out than before? | Sliding scale 0-100 with following labels: Much less left out (0-12.5) – Slightly less (12.6-37.5) – The same amount (37.6-62.5) – Slightly more (62.6-87.5) – Much more left out (87.6-100) |
| Loneliness (reference) | How often do you feel lonely? | Hardly ever or never – Some of the time – Often |
| Loneliness (change during lockdown) | During lockdown, have you felt less, the same or more lonely than before? | Sliding scale 0-100 with following labels: Much less lonely (0-12.5) – Slightly less (12.6-37.5) – The same amount (37.6-62.5) – Slightly more (62.6-87.5) – Much more lonely 87.6-100) |
| Concern about appearance** | *Some young people feel worried about things happening around them. To what extent do you worry about the following topics:*  My appearance (how I look and what I wear) | Sliding scale 0-100 with following labels: Not at all worried (0-12.5) – Not very worried (12.6-37.5) – Quite worried (37.6-62.5) – Worried (62.6-87.5) – Extremely worried (87.6-100) |
| **Lifestyle factors** | | |
| Exercise (left house during lockdown) | During lockdown, have you left the house for exercise (e.g. walk/run/cycle/skate)? | Sliding scale 0-100 with following labels: Not at all (0-12.5) – Once or twice (12.6-37.5) – Sometimes (37.6-62.5) – Most days (62.6-87.5) – Every day (87.6-100) |
| Exercise (change during lockdown) | During lockdown, have you done less, the same, or more exercise than before? | Sliding scale 0-100 with following labels: Much less (0-12.5) – Slightly less (12.6-37.5) – The same amount (37.6-62.5) – Slightly more (62.6-87.5) – Much more (87.6-100) |
| Sleep (change during lockdown) | During lockdown, has your sleep been worse, the same or better than before? | Sliding scale 0-100 with following labels: Much worse (0-12.5) – Slightly worse (12.6-37.5) – The same (37.6-62.5) – Slightly better (62.6-87.5) – Much better (87.6-100) |
| **Return to school** | | |
| Seeing friends again | Thinking about going back to school when lockdown ends, how do you feel about the following?  Seeing friends again | Sliding scale 0-100 with following labels: Dreading it (0-12.5) – Slightly worried (12.6-37.5) – Neutral (37.6-62.5) – Looking forward to it (62.6-87.5) – Can’t wait (87.6-100) |
| Seeing other classmates/peers | Thinking about going back to school when lockdown ends, how do you feel about the following?  Seeing other classmates | Sliding scale 0-100 with following labels: Dreading it (0-12.5) – Slightly worried (12.6-37.5) – Neutral (37.6-62.5) – Looking forward to it (62.6-87.5) – Can’t wait (87.6-100) |
| School work | Thinking about going back to school when lockdown ends, how do you feel about the following?  School work | Sliding scale 0-100 with following labels: Dreading it (0-12.5) – Slightly worried (12.6-37.5) – Neutral (37.6-62.5) – Looking forward to it (62.6-87.5) – Can’t wait (87.6-100) |
| Attending lessons | Thinking about going back to school when lockdown ends, how do you feel about the following?  Attending lessons | Sliding scale 0-100 with following labels: Dreading it (0-12.5) – Slightly worried (12.6-37.5) – Neutral (37.6-62.5) – Looking forward to it (62.6-87.5) – Can’t wait (87.6-100) |
| Being away from home | Thinking about going back to school when lockdown ends, how do you feel about the following?  Being away from home | Sliding scale 0-100 with following labels: Dreading it (0-12.5) – Slightly worried (12.6-37.5) – Neutral (37.6-62.5) – Looking forward to it (62.6-87.5) – Can’t wait (87.6-100) |
| Sports and exercise activities | Thinking about going back to school when lockdown ends, how do you feel about the following?  Sports and exercise activities | Sliding scale 0-100 with following labels: Dreading it (0-12.5) – Slightly worried (12.6-37.5) – Neutral (37.6-62.5) – Looking forward to it (62.6-87.5) – Can’t wait (87.6-100) |
| Other school and/or outside-school clubs | Thinking about going back to school when lockdown ends, how do you feel about the following?  Other school and/or outside-school clubs | Sliding scale 0-100 with following labels: Dreading it (0-12.5) – Slightly worried (12.6-37.5) – Neutral (37.6-62.5) – Looking forward to it (62.6-87.5) – Can’t wait (87.6-100) |
| Travelling to and from school | Thinking about going back to school when lockdown ends, how do you feel about the following?  Travelling to and from school | Sliding scale 0-100 with following labels: Dreading it (0-12.5) – Slightly worried (12.6-37.5) – Neutral (37.6-62.5) – Looking forward to it (62.6-87.5) – Can’t wait (87.6-100) |

*For our analyses, we transformed all variables originally measured on a 0-100 sliding scale into the categorical variables indicated by the survey labels.

**Asked only of secondary school and FEC students
