# Supplementary material for: Happier during lockdown: a descriptive analysis of self-reported wellbeing in 17,000 UK school students during Covid-19 lockdown
Source: Eur Child Adolesc Psychiatry. 2022 Feb 17;32(6):1131–46. doi: 10.1007/s00787-021-01934-z (PMC8853175; doi:10.1007/s00787-021-01934-z)
Supplement: Supplementary file 5 — Supplementary file5 (DOCX 17 KB) [file 787_2021_1934_MOESM5_ESM.docx]

**Supplementary Table 3.** Feelings about returning to school post-lockdown

| **Variable** | **Missing data (%)** | **Worse *N (%)*** | **The Same *N (%)*** | **Better *N (%)*** |
| --- | --- | --- | --- | --- |
| **Return to school** | | | | |
| **Seeing friends again** | 1.9 |  |  |  |
| Can't wait |  | 2445 (48.4) | 2638 (52.8) | 2352 (48.3) |
| Looking forward to it |  | 1476 (29.2) | 1589 (31.8) | 1622 (33.3) |
| Neutral |  | 403 (8.0) | 510 (10.2) | 527 (10.8) |
| Slightly worried |  | 506 (10.0) | 198 (4.0) | 260 (5.3) |
| Dreading it |  | 221 (4.4) | 58 (1.2) | 108 (2.2) |
| **Seeing other classmates/peers** | 3.3 |  |  |  |
| Can't wait |  | 1056 (21.1) | 1153 (23.3) | 1042 (21.8) |
| Looking forward to it |  | 1730 (34.6) | 2157 (43.5) | 1858 (38.8) |
| Neutral |  | 1134 (22.7) | 1262 (25.5) | 1273 (26.6) |
| Slightly worried |  | 666 (13.3) | 244 (4.9) | 371 (7.8) |
| Dreading it |  | 410 (8.2) | 138 (2.8) | 239 (5.0) |
| **Schoolwork** | 4.5 |  |  |  |
| Can't wait |  | 305 (6.2) | 283 (5.8) | 340 (7.2) |
| Looking forward to it |  | 1087 (21.9) | 1188 (24.3) | 1161 (24.5) |
| Neutral |  | 1434 (28.9) | 2301 (47.1) | 1890 (39.8) |
| Slightly worried |  | 1187 (24.0) | 702 (14.4) | 835 (17.6) |
| Dreading it |  | 942 (19.0) | 412 (8.4) | 521 (11.0) |
| **Attending lessons** | 4.7 |  |  |  |
| Can't wait |  | 544 (11.0) | 522 (10.7) | 546 (11.5) |
| Looking forward to it |  | 1456 (29.4) | 1638 (33.4) | 1562 (33.0) |
| Neutral |  | 1438 (29.0) | 2056 (42.0) | 1743 (36.9) |
| Slightly worried |  | 898 (18.1) | 468 (9.6) | 525 (11.1) |
| Dreading it |  | 619 (12.5) | 213 (4.3) | 353 (7.5) |
| **Being away from home** | 6.7 |  |  |  |
| Can't wait |  | 908 (18.5) | 449 (9.3) | 397 (8.6) |
| Looking forward to it |  | 1418 (28.9) | 1088 (22.6) | 993 (21.6) |
| Neutral |  | 1746 (35.5) | 2824 (58.8) | 2364 (51.4) |
| Slightly worried |  | 573 (11.7) | 332 (6.9) | 599 (13.0) |
| Dreading it |  | 269 (5.5) | 113 (2.4) | 247 (5.4) |
| **Sports and exercise activities** | 4.0 |  |  |  |
| Can't wait |  | 1429 (28.9) | 1557 (31.6) | 1456 (30.6) |
| Looking forward to it |  | 1480 (29.9) | 1680 (34.1) | 1648 (34.6) |
| Neutral |  | 1261 (25.5) | 1349 (27.4) | 1220 (25.6) |
| Slightly worried |  | 424 (8.6) | 178 (3.6) | 242 (5.1) |
| Dreading it |  | 350 (7.1) | 159 (3.2) | 199 (4.2) |
| **Other school and/or outside-school clubs** | 6.2 |  |  |  |
| Can't wait |  | 1343 (27.8) | 1519 (31.3) | 1410 (30.5) |
| Looking forward to it |  | 1290 (26.7) | 1469 (30.3) | 1420 (30.7) |
| Neutral |  | 1621 (33.5) | 1636 (33.7) | 1483 (32.0) |
| Slightly worried |  | 336 (7.0) | 139 (2.9) | 191 (4.1) |
| Dreading it |  | 243 (5.0) | 92 (1.9) | 125 (2.7) |
| **Travelling to and from school** | 6.6 |  |  |  |
| Can't wait |  | 571 (11.8) | 562 (11.6) | 613 (13.2) |
| Looking forward to it |  | 1172 (24.1) | 1208 (25.0) | 1207 (26.0) |
| Neutral |  | 2085 (42.9) | 2563 (53.1) | 2150 (46.2) |
| Slightly worried |  | 649 (13.4) | 344 (7.1) | 402 (8.6) |
| Dreading it |  | 379 (7.8) | 154 (3.2) | 278 (6.0) |
